# Sexual Activity and Hepatitis B and C Virus Infection Among Young Adults After Introduction of a Vaccination Program in an Area of High Endemicity

**DOI:** 10.2188/jea.JE20081010

**Published:** 2009-09-05

**Authors:** Min Kyung Lim, Silvia Franceschi, Salvatore Vaccarella, Young-Hee Ju, Jin-Kyoung Oh, Hyun-Joo Kong, Dong-Il Kim, Byoung-Gwon Kim, Jung-Il Kim, Kap-Yeol Jung, Dong-Soon Lee, Hai-Rim Shin

**Affiliations:** 1 National Cancer Control Research Institute, National Cancer Center, Goyang, Republic of Korea; 2 International Agency for Research on Cancer, 150 cours Albert Thomas, 69372 Lyon cedex 08, France; 3 Samsung Medical Center, Sungkunkwan University School of Medicine, Suwon, Republic of Korea; 4 College of Medicine, Dong-A University, Busan, Republic of Korea; 5 College of Medicine, Seoul National University, Seoul, Republic of Korea

**Keywords:** hepatitis B virus, hepatitis C virus, sexual transmission, vaccination, endemic area

## Abstract

**Background:**

In areas where hepatitis is endemic, little is known about the sexual transmission of HBV after introduction of an HBV vaccination program.

**Methods:**

We used a self-administered questionnaire and serological tests for HBsAg, anti-HBs, anti-HBc, and anti-HCV to examine the role of sexual activity, as well as sociodemographic status, lifestyle habits, and a history of vaccinations, transfusions, and surgery, in the transmission of HBV and HCV in Korea. The subjects were 865 female and 541 male university students (median age, 19 years; age range, 16–25).

**Results:**

Overall seropositivity was 8.1% for HBsAg, 69.3% for anti-HBs, 21.3% for anti-HBc, and 0.4% for anti-HCV. Regarding HBV, 8% of the subjects were chronic carriers or had recently been infected, 22.8% were never exposed and nonvaccinated, 16.6% were exposed noncarriers, and 52.7% had most likely been vaccinated. We found a significant association between HBsAg seropositivity and history of sexual intercourse (Odds Ratio, 1.8; 95% CI, 1.1–2.8). Students without serologic evidence of immunization against HBV were more likely to have become HBsAg-positive after becoming sexually active.

**Conclusions:**

Our findings suggest that sexual transmission does occur among adolescents and young adults who have not been vaccinated, whereas vaccination protects individuals from becoming an HBV carrier after becoming sexually active.

## INTRODUCTION

Hepatitis B virus (HBV) is associated with acute and chronic liver disease, including fatal liver cancer, and is a worldwide public health problem. In areas of endemic infection, the World Health Organization (WHO) strongly recommends universal vaccination with a plasma-derived vaccine. The HBV vaccine, which has been available since 1982, is approximately 95% effective in preventing chronic infection.^1^

In Korea, the HBV vaccine was introduced in 1983. It was integrated into the routine childhood immunization schedule to address the problem of chronic HBV infection in 1995,^2^ at which time Korea had one of the highest incidences of liver cancer in the world.^3^ The program led to a dramatic decrease in hepatitis B surface antigen (HBsAg) seropositivity. In 2001, 4.4% of the overall population (male, 4.7%; female, 4.2%) were seropositive, and the rate was 2% among children and adolescents born from 1982 through 1991 (male, 2.5%; female, 1.2%).^4^ Nevertheless, HBsAg seropositivity was still relatively high in adults aged 20–29 years, who were born from 1972 through 1981 (overall, 4.9%; male, 5.6%, female, 4.4%).^4^ This suggests that HBV infection is acquired at a young age through parenteral and/or sexual transmission, and through perinatal transmission.^5^

In 2004, we reported an HBsAg seropositivty rate of 8.1% in university students born in the period from 1979 to 1983 (median year of birth, 1982; standard deviation of birth year, 2.4) in Busan^6^—a city with both a high incidence of liver cancer^2^ and, not surprisingly, a high prevalence (5%–7.4%) of HBV infection among adults.^7^^,^^8^

Here we defined HBV infection status by using serologic findings from 3 different markers (HBsAg, antibody to hepatitis B surface antigen [anti-HBs], and hepatitis B core antigen antibody [anti-HBc]) to assess the risk of heterosexual HBV transmission and to determine whether HBV vaccination was effective against such transmission. We also tested for anti-HCV to examine the controversial possibility that it is sexually transmitted.^9^^,^^10^

## METHODS

### Subjects

We conducted the present survey between 29 August and 30 September 2002 in Busan, Korea to evaluate the prevalence of genital papillomavirus infection with respect to sexual habits in male and female adolescents and young adults. Our study methods have been previously reported.^11^ Briefly, we contacted male and female students from 3 institutions of higher education and arranged meetings of 30 to 70 students on the university premises. At each meeting, our study team (1 male and 1 female physician, 2 nurses, 1 laboratory technician, and 1 research assistant) gave a brief presentation on various issues concerning health education and disease prevention. Then the objectives of the present survey were explained before obtaining informed consent and collecting the information for the survey. Students were asked to complete, in private, a self-administered questionnaire that inquired about sociodemographic status; lifestyle habits; and history of vaccinations, transfusions, surgery, and sexual activity (whether they had engaged in penetrative sexual intercourse, age at first intercourse, and number of sexual partners). Each study participant returned, in a sealed envelope, a coded but anonymous questionnaire.

Finally, we contacted a total of 900 female and 600 male students; 865 (96.1%) of the women and 541 (90.2%) of the men agreed to participate in interviews and serologic examinations. Their median age was 19 years, and 88.0% of them were between 17 and 25 years old (range, 16–25).

Each student’s desire to participate in this study and to provide a blood sample was confirmed by an explicit statement on an informed consent form. This form also explained the confidentiality of private information and that the subject could refuse to participate or withdraw from the study at any time, without penalty or loss of accrued benefits. The informed consent form was signed by all subjects and the study protocol was approved by the institutional review board of the National Cancer Center of Korea.

### Serological study

We collected 2 blood samples from each participant: one in a 3-mL tube containing EDTA and one in a 10-mL heparinized vacutainer (Becton Dickinson, Franklin Lakes, NJ, USA). The tubes were sent in iceboxes on the day of collection to the laboratory of Dong-A University Hospital, where the AxSYM system (Abbott, Abbott Park, IL, USA) was used for screening. HBsAg (V2) and AUSAB were used to test for HBsAg and anti-HBsAg. Anti-HBcAg (IgG) was tested with CORE and CORE-M (Ig-M), which was performed on samples that were positive on the IgG test.

Sera were screened for antibodies against HCV using AxSYM HCV version 3.0 (Abbott, Abbott Park, IL, USA).

### Statistical analysis

Unconditional logistic regression analysis including age group and sex was used to compute odds ratios (ORs) and corresponding 95% confidence intervals (CIs) for HBsAg seropositivity and for being sexually active. We evaluated linear trends for the risk of being positive for HBsAg according to the number of sexual partners or years since the first intercourse by likelihood ratio tests for trend based on linear logistic regression models.

We divided the subjects into 4 groups based on HBV serologic markers: 1) carrier or recently infected, 2) unexposed and not vaccinated, 3) exposed but noncarrier, and 4) vaccinated.^12^ We then computed the OR for sexual activity across the different serological groups to explore the possible consequences of recent sexual exposure to HBV in nonvaccinated versus vaccinated students.

## RESULTS

Overall seropositivity was 8.1% for HBsAg, 69.3% for anti-HBs, 21.3% for anti-HBc, and 0.4% for Anti-HCV. Table 1 shows the prevalence of the serological markers for HBV overall and by gender. A total of 722 students (52.7%) and 110 students (8.0%) were included in the vaccinated group and the carrier or recently infected group, respectively.

**Table 1.** Prevalence of serological markers for HBV among the study population, by gender

Serological marker				Male (*n* = 530)	Female (*n* = 841)	Total (*n* = 1371^c^)
			
HBsAg	Anti-HBs	Anti-HBc	Definition	*n* (%)	*n* (%)	*n* (%)
+	+/−	+/−	Carrier or recently infected^a^	41 (7.7)	69 (8.2)	110 (8.0)
−	−	−	Unexposed nonvaccinated	114 (21.5)	198 (23.5)	312 (22.8)
−	+/−	+	Exposed noncarrier^b^	98 (18.5)	129 (15.3)	227 (16.6)
−	+	−	Vaccinated	277 (52.3)	445 (52.9)	722 (52.7)

HBV = hepatitis B virus; HBsAg = hepatitis B virus surface antigen; Anti-HBs = hepatitis B virus surface antibody; Anti-HBc = hepatitis B virus core antibody; − and + indicate negative and positive, respectively.

^a^Comprising 54.6% of subjects who were HBsAg (+), Anti-HBs (−), Anti-HBc (+); 24.5% who were HBsAg (+), Anti-HBs (−), Anti-HBc (−); 16.4% who were HBsAg (+), Anti-HBs (+), Anti-HBc (−); and 4.5% who were HBsAg (+), Anti-HBs (+), Anti-HBc (+).

^b^Comprising of subjects who were 93.8% HBsAg (−), Anti-HBs (+), Anti-HBc (+); and 6.2% who were HBsAg (−), Anti-HBs (−), Anti-HBc (+).

^c^Subjects who had test results for all 3 serologic markers of HBV.

Table 2 shows the association between HBsAg seropositivity and gender, age group, and other selected characteristics. Seropositivity increased up to the age of 18–19 and then reached a plateau. A significant association between seropositivity and a history of sexual intercourse was observed. There was no trend in the risk of HBsAg positivity with respect to the number of sexual partners or years since first intercourse.

**Table 2.** Odds ratios for HBsAg seropositivity, by selected characteristics

Variables		No. of subjects^a^	HBsAg-positive No. (%)	Adjusted OR^b^ (95% CI)
Gender	Male^c^	536	41 (7.7)	1 (reference)
	Female	853	71 (8.3)	1.24 (0.82–1.90)
Age group	<18 years^c^	128	3 (2.3)	1 (reference)
	18–19 years	625	56 (9.0)	4.34 (1.33–14.16)
	≥20 years	636	53 (8.3)	4.29 (1.29–14.16)
Smoking status	Never^c^	714	54 (7.6)	1 (reference)
	Ever	516	44 (8.5)	1.27 (0.81–1.99)
Alcohol drinking	Never^c^	409	31 (7.6)	1 (reference)
	Ever	895	76 (8.5)	1.05 (0.67–1.64)
Self-reported	Never^c^	179	19 (10.6)	1 (reference)
HBV vaccination	Ever	431	38 (8.8)	0.70 (0.39–1.27)
	Unknown	779	55 (7.1)	0.56 (0.32–0.99)
Surgery	Never^c^	919	72 (7.8)	1 (reference)
	Ever	318	28 (8.8)	1.12 (0.71–1.78)
Acupuncture	Never^c^	784	65 (8.3)	1 (reference)
	Ever	510	38 (7.5)	0.86 (0.56–1.31)
Blood transfusion	Never^c^	1256	102 (8.1)	1 (reference)
	Ever	57	6 (10.5)	1.31 (0.54–3.14)
Sexual intercourse	No^c^	739	45 (6.1)	1 (reference)
	Yes	460	44 (9.6)	1.76 (1.11–2.79)
	Not reported	190	23 (12.1)	2.40 (1.40–4.10)
Number of	1^c^	148	12 (8.1)	1 (reference)
sexual partners^d^	2–3	136	14 (10.3)	1.52 (0.66–3.46)
	≥4	164	15 (9.2)	1.38 (0.60–3.19)
			*χ^2^ for trend*	0.27; *P* = 0.598
Years since first	<1^c^	124	13 (10.5)	1 (reference)
intercourse^d^	1–2	125	15 (12.0)	1.21 (0.54–2.70)
	≥3	200	15 (7.5)	0.80 (0.33–1.96)
			*χ^2^ for trend*	0.67; *P* = 0.411

HBsAg = hepatitis B virus surface antigen; OR = odds ratio; 95% CI = 95% confidence interval.

^a^Some strata do not sum to the total because of missing values.

^b^Odds ratio using logistic regression model adjusted for gender and age group, as appropriate.

^c^Reference category.

^d^Only sexually active subjects.

A history of surgery, acupuncture, and blood transfusion were all unrelated to HBsAg positivity. Many students were not sure whether they had received the HBV vaccine, and the frequency of HBsAg-positivity was not significantly lower in those who reported being vaccinated than in those who did not.

Because age and history of sexual intercourse were strongly correlated, and both factors were associated with HBsAg positivity, we examined the association between history of sexual intercourse and HBsAg positivity in different age strata (Table 3). ORs of HBsAg positivity were higher among subjects aged 18 or older.

**Table 3.** Odds ratios for HBsAg seropositivity in each age strata, according to sexual intercourse status

Age (years)	<18			18–19			≥20		

Sexual intercourse	No. of subjects	HBsAg-positive No. (%)	Adjusted OR^a^ (95% CI)	No. of subjects^a^	HBsAg-positive No. (%)	Adjusted OR^a^ (95% CI)	No. of subjects^a^	HBsAg-positive No. (%)	Adjusted OR^a^ (95% CI)
No	70	1 (1.4)	1 (reference)	409	30 (7.4)	1 (reference)	260	14 (5.4)	1 (reference)
Yes	20	0 (0.0)	—	132	17 (12.9)	1.90 (1.01–3.57)	308	27 (8.8)	1.88 (0.93–3.79)
Not reported	38	2 (5.3)	3.83 (0.34–43.72)	84	9 (10.7)	1.50 (0.68–3.30)	68	12 (17.7)	3.89 (1.70–8.89)

HBsAg = hepatitis B virus surface antigen; OR = odds ratio; 95% CI = 95% confidence interval.

^a^Odds ratio using logistic regression model adjusted for gender.

Among nonvaccinated subjects who were defined by serological testing, sexually active subjects had a 49% increased risk of HBsAg positivity (OR, 1.49; 95% CI, 0.92–2.45). There was a significantly higher risk (OR, 1.76; 95% CI, 1.00–3.12) among students with unreported sexual activity (Table 4)

**Table 4.** Odds ratios for HBsAg seropositivity among the nonvaccinated group, according to sexual intercourse status

Variable		No. of subjects^a^	HBsAg-positive No. (%)	Adjusted OR^b^ (95% CI)
Sexual	No	309	44 (14.2)	1 (reference)
intercourse	Yes	229	43 (18.8)	1.49 (0.92–2.45)
	Not reported	111	23 (20.7)	1.76 (1.00–3.12)

HBsAg = hepatitis B virus surface antigen; OR = odds ratio; 95% CI = 95% confidence interval.

^a^Vaccination status of the nonvaccinated group was determined serologically.

^b^Odds ratio using logistic regression model adjusted for age and gender.

Among the 5 students who were anti-HCV-positive, 1 reported a history of surgery and 2 of acupuncture. Some reported no history of penetrative sexual intercourse and none was positive for HBsAg (Appendix).

**Appendix.** Anti-HCV positive subjects and risk factors

Subject No	Anti-HCV titer	Age (Years)	Gender	HBsAg	Anti-HBc	Anti-HBs	Sexual activity	Blood Transfusion history
1	1.10	17	Female	Negative	Negative	Negative	Unknown	Never
2	2.82	17	Female	Negative	Negative	Positive	Unknown	Never
3	1.64	18	Male	Negative	Negative	Negative	Inactive	Never
4	1.26	18	Female	Negative	Negative	Positive	Inactive	Never
5	1.43	18	Female	Negative	Negative	Negative	Unknown	Never

Anti-HCV = Antibody to hepatitis C virus; HBsAg = hepatitis B virus surface antigen; Anti-HBs = hepatitis B virus surface antibody; Anti-HBc = hepatitis B virus core antibody.

## DISCUSSION

**Figure 1.**
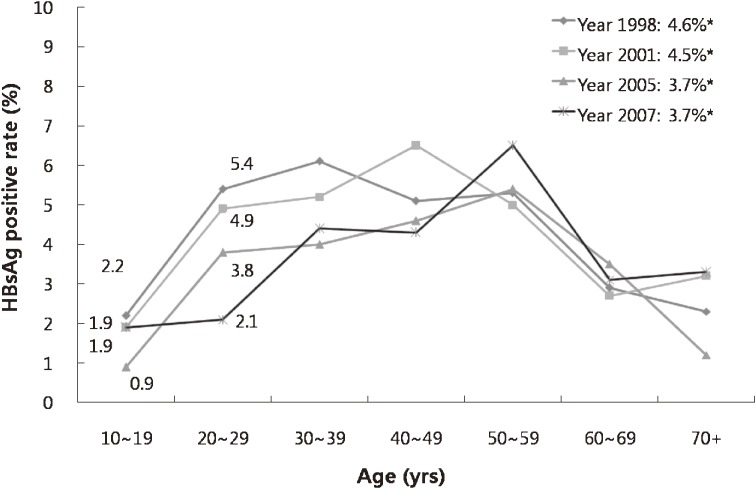
HBsAg seropositivity in Korea by year and age (Data from ‘National Health and Nutrition Survey’ in Korea, 1998, 2001, 2004, 2007). HBsAg = hepatitis B virus surface antigen; yrs = years. *Overall positive rate among persons aged over 10 years.

In Korea, after the introduction of the HBV vaccine in 1983, the first national HBV vaccination program for newborn infants whose mothers were HBsAg carriers began in 1985. The program was extended to all health insurance beneficiaries and school children in 1988, and all newborn infants in 1991,^13^ but coverage remained incomplete. In 1995, HBV vaccination was integrated into the routine childhood immunization schedule.^2^ As a result, Korea changed from high to intermediate endemicity and overall HBsAg seropositivity decreased rapidly among individuals younger than 20 years, ie, those gradually reached by HBV vaccination (Figure 1).^4^^,^^14^^–^^16^ By 2001, a decline in HBsAg positivity started to emerge among young adults, too, and by 2005, the national survey showed that HBsAg positivity had declined in all age groups up to 40–49 years of age.

Our findings showed that, in a population not yet fully vaccinated, sexual transmission could increase HBV infection among young people (OR = 1.8; CI: 1.1–2.8). HBsAg seropositivity did differ between age groups: people aged 18 years or older had a level (9.0%) that was approximately 4-fold higher than that of subjects younger than 18 years. In addition, a positive association between history of sexual intercourse and HBsAg positivity was found in subjects aged 18 years or older. Similar patterns are observed in areas not endemic for HBV, where no mass vaccination is in place, and where sexual transmission is the major infection route. In such areas, the incidence of HBV infection increases markedly starting at 15–19 years of age (the usual time of first sexual intercourse) and reaches a peak at 20–24 years of age, after which it steadily decreases.^17^^,^^18^

The attribution of sexual or parenteral transmission of HBV is expected to be relatively higher, as infant immunization programs reduce the number of prenatal and childhood infections. In Japan—an area of intermediate HBV endemicity—the main route of HBV transmission has changed from perinatal to sexual transmission during the last decade.^19^ In areas of high endemicity, where perinatal infection is common, sexual contact is classified as an additional risk factor and the level of risk varies with the study population.^10^^,^^20^^,^^21^

The overall HBsAg seropositivity in the present study is higher than those reported in other recent studies in Korea.^22^^,^^23^ The high levels of HBsAg in Busan can be explained by the large reservoir of chronically infected persons in regions where HBV was endemic and where effective immunization programs are yet to reach the majority of people.^22^ Although HBsAg seropositivity did not significantly differ between males and females in the present study, our observation of higher seropositivity among females contradicts the findings of other studies^22^^,^^23^ and requires further evaluation.

Our results do not support the findings of studies that reported a history of surgery, acupuncture, and blood transfusion as significant risk factors among young Koreans.^5^^,^^7^^,^^8^ In addition, smoking and alcohol consumption were not associated with HBsAg seropositivity, although they were strongly associated with sexual activity.

Our findings regarding anti-HCV seropositivity in the same population provides baseline information for future study of HCV infection in adolescents and young adults in Korea, although we were unable to estimate the effect of sexual intercourse on HCV infection due to ambiguous information on sexual exposure and the small number of cases.

Our study provides valuable information on HBV infection status and the possibility of sexual transmission among young adults in an endemic area. We had access to a large number of students at their school, and an open, close collaboration with both the university authorities and the Public Health Welfare and Women’s Affairs Bureau of Metropolitan Busan. In addition, a well organized expert team—which provided education on health issues and explanations of the purpose and importance of this study—played a major role in helping students understand the study goal. We also included comprehensive information on the informed consent forms to assure the confidentiality of the self-administered questionnaire. These planned field approaches led to relatively high participation from the targeted student population, including an agreement to provide a blood sample. Many previous studies had difficulties in achieving a high level of cooperation from this population. These factors contributed to our obtaining meaningful results in this study.

There were some limitations to our study, however. Self-reporting of HBV vaccination was not accurate, because more than half of the students did not remember whether they had been vaccinated. However, it was possible to use serological findings to compensate for the missing information on the vaccination history. Because those who did and did not respond to the question on sexual activity had similar demographic and behavioral characteristics, and because nonrespondents had a stronger association with HBsAg positivity (OR = 2.4; CI: 1.4–4.1) than did sexually active respondents, we believe that excluding cases where sexual activity was unreported did not modify the study results or lead to an overestimation of the association between sexual activity and HBsAg seropositivity. Regarding the analysis of the nonvaccinated group, because sexual activity was associated with HBsAg positivity, and this association was stronger when sexually active subjects were aggregated with subjects with unreported sexual activity, we believe that sexual transmission did occur, and was more frequent, among subjects who did not report their sexual experience.

We did not collect information on vertical transmission and family history of HBV infection in this study. It was also impossible to distinguish between chronic and newly acquired HBV infection because we determined the prevalence of HBsAg seropositivity at a single time point. In addition, among the HBsAg-positive sexually active students, 25 (58.1%) were also positive for anti-HBc and 5 (11.4%) were positive for anti-HBsAg (with or without anti-HBc). However, this does not affect the association between sexual activity and HBV infection (the main result of this study), if we assume that the probability of vertical transmission is the same for the sexually active and inactive groups.

Despite these limitations, our results suggest that, in an endemic area, sexual transmission might be an additional route of HBV infection among adolescents and young adults who have not been vaccinated, whereas vaccinated individuals are protected from becoming chronic HBV carriers after becoming sexually active. However, in Korea and other endemic areas, we must continue to monitor the changing patterns of transmission in order to assist nationwide HBV vaccination programs in implementing effective prophylactic policies.
